# Correction: Rural to Urban Population Density Scaling of Crime and Property Transactions in English and Welsh Parliamentary Constituencies

**DOI:** 10.1371/journal.pone.0167605

**Published:** 2016-12-02

**Authors:** Quentin S. Hanley, Dan Lewis, Haroldo V. Ribeiro

Eq 3 and the subsequent inline equation are incorrect. The correct equation and inline equation should be: “

$$\log\;y=\{\begin{array}{l} \log\;y_{0}+\beta_{L}\log\;d\;for\;\left(d<d^{*}\right)\\ \log\;y_{1}+\beta_{H}\log\;d\;for\;\left(d\geq d^{*}\right) \end{array},$$ (3)

where d* is a population density threshold, *β*_L_ (*β*_H_) is the power-law exponent for low (high) population density, *y*_0_ and *y*_1_ are constants. In particular, we have chosen log *y*_1_ = log *y*_0_ + (*β*_*L*_ − *β*_*H*_)log d^*^, holding the continuity of *y*(*d*).”
