# Supplementary material for: Rural to Urban Population Density Scaling of Crime and Property Transactions in English and Welsh Parliamentary Constituencies
Source: PLoS One. 2016 Feb 17;11(2):e0149546. doi: 10.1371/journal.pone.0149546 (PMC4757021; doi:10.1371/journal.pone.0149546)
Supplement: S1 Fig — Similarly to adjusted R2, markedly improved correlations are observed using density metrics which were superior in all cases. Here the error bars stand for 99% confidence interval obtained via bootstrap. Unlike adjusted R2, MIC indicates no significant difference between population density and day population density (via bootstrap two-sample mean test with 99% confidence) for other theft and shoplifting. (PDF) [file pone.0149546.s002.pdf]

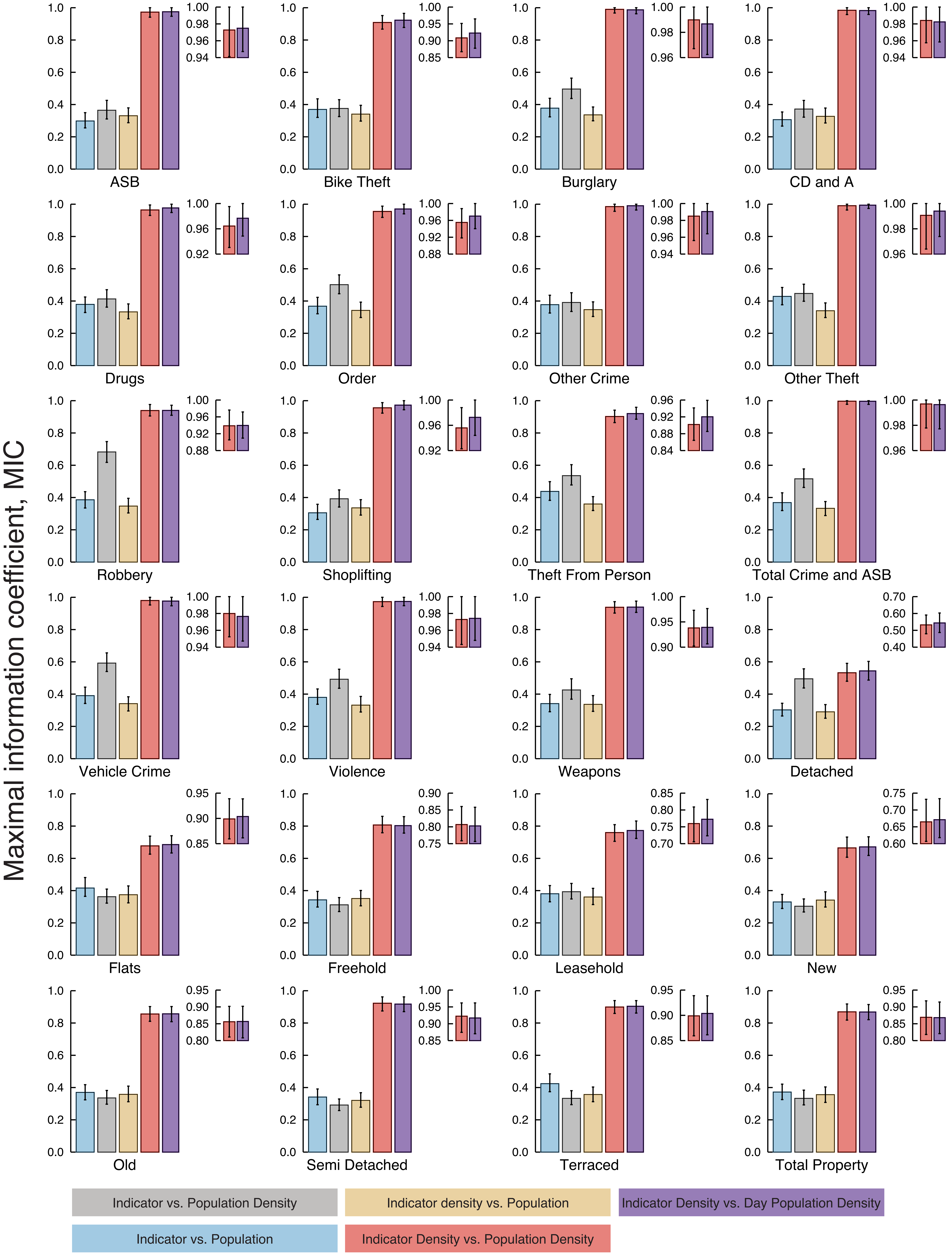
Maximal information coefficient, MIC
ASB
Bike Theft
Burglary
CD and A
Drugs
Order
Other Crime
Other Theft
Robbery
Shoplifting
Theft From Person
Total Crime and ASB
Vehicle Crime
Violence
Weapons
Detached
Flats
Freehold
Leasehold
New
Old
Semi Detached
Terraced
Total Property
Indicator vs. Population Density
Indicator density vs. Population
Indicator Density vs. Day Population Density
Indicator vs. Population
Indicator Density vs. Population Density
