# Supplementary material for: Rural to Urban Population Density Scaling of Crime and Property Transactions in English and Welsh Parliamentary Constituencies
Source: PLoS One. 2016 Feb 17;11(2):e0149546. doi: 10.1371/journal.pone.0149546 (PMC4757021; doi:10.1371/journal.pone.0149546)
Supplement: S3 Fig — Error bars stand for 99% bootstrap confidence intervals and the asterisk marks indicate a significant difference (via bootstrap two-sample mean test with 99% confidence). Notice that the BIC criteria differs from the adjusted R2 only for ASB, bike theft and freehold. (PDF) [file pone.0149546.s004.pdf]

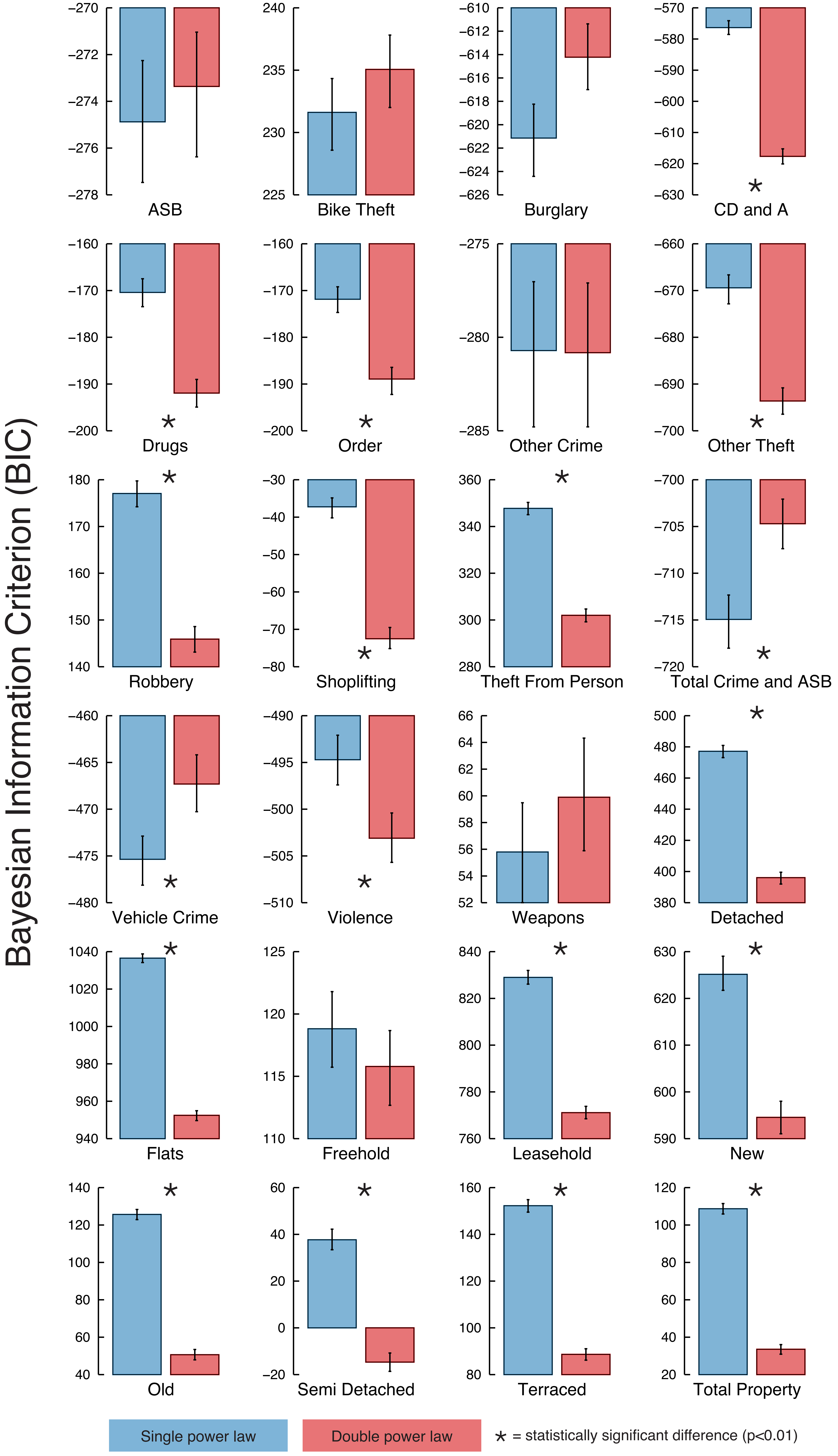
Bayesian Information Criterion (BIC)
ASB
Bike Theft
Burglary
CD and A
Drugs
Order
Other Crime
Other Theft
Robbery
Shoplifting
Theft From Person
Total Crime and ASB
Vehicle Crime
Violence
Weapons
Detached
Flats
Freehold
Leasehold
New
Old
Semi Detached
Terraced
Total Property
Single power law
Double power law
* = statistically significant difference (p<0.01)
