# Supplementary material for: Rural to Urban Population Density Scaling of Crime and Property Transactions in English and Welsh Parliamentary Constituencies
Source: PLoS One. 2016 Feb 17;11(2):e0149546. doi: 10.1371/journal.pone.0149546 (PMC4757021; doi:10.1371/journal.pone.0149546)
Supplement: S4 Fig — These three metrics were the only cases where the criteria diverged. These can be considered marginal cases of urban scaling transitions. (PDF) [file pone.0149546.s005.pdf]

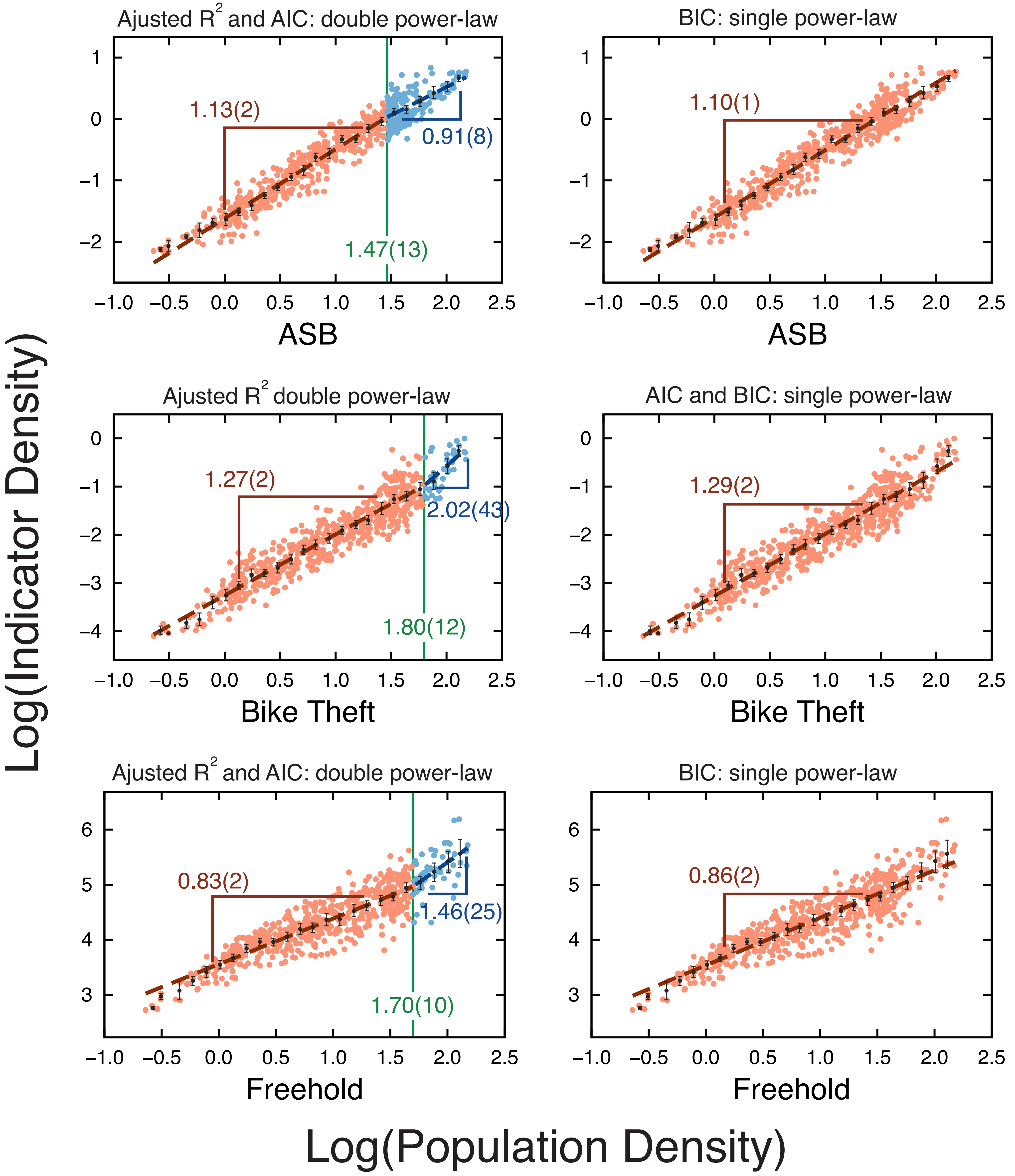

Ajusted R² and AIC: double power-law
1.13(2)
0.91(8)
1.47(13)
ASB
BIC: single power-law
1.10(1)
ASB
Ajusted R² double power-law
1.27(2)
2.02(43)
1.80(12)
Bike Theft
AIC and BIC: single power-law
1.29(2)
Bike Theft
Ajusted R² and AIC: double power-law
0.83(2)
1.46(25)
1.70(10)
Freehold
BIC: single power-law
0.86(2)
Freehold
Log(Indicator Density)
Log(Population Density)
